# Optimizing the Distribution of Leg Muscles for Vertical Jumping

**DOI:** 10.1371/journal.pone.0150019

**Published:** 2016-02-26

**Authors:** Jeremy D. Wong, Maarten F. Bobbert, Arthur J. van Soest, Paul L. Gribble, Dinant A. Kistemaker

**Affiliations:** 1 Brain and Mind Institute, Western University, Ontario, Canada; 2 MOVE Research Institute, Vrije Universiteit Amsterdam, Nord-Holland, The Netherlands; 3 Biomedical Physiology and Kinesiology, Simon Fraser University, British Columbia, Canada; University of Zurich, SWITZERLAND

## Abstract

A goal of biomechanics and motor control is to understand the design of the human musculoskeletal system. Here we investigated human functional morphology by making predictions about the muscle volume distribution that is optimal for a specific motor task. We examined a well-studied and relatively simple human movement, vertical jumping. We investigated how high a human could jump if muscle volume were optimized for jumping, and determined how the optimal parameters improve performance. We used a four-link inverted pendulum model of human vertical jumping actuated by Hill-type muscles, that well-approximates skilled human performance. We optimized muscle volume by allowing the cross-sectional area and muscle fiber optimum length to be changed for each muscle, while maintaining constant total muscle volume. We observed, perhaps surprisingly, that the reference model, based on human anthropometric data, is relatively good for vertical jumping; it achieves 90% of the jump height predicted by a model with muscles designed specifically for jumping. Alteration of cross-sectional areas—which determine the maximum force deliverable by the muscles—constitutes the majority of improvement to jump height. The optimal distribution results in large vastus, gastrocnemius and hamstrings muscles that deliver more work, while producing a kinematic pattern essentially identical to the reference model. Work output is increased by removing muscle from rectus femoris, which cannot do work on the skeleton given its moment arm at the hip and the joint excursions during push-off. The gluteus composes a disproportionate amount of muscle volume and jump height is improved by moving it to other muscles. This approach represents a way to test hypotheses about optimal human functional morphology. Future studies may extend this approach to address other morphological questions in ethological tasks such as locomotion, and feature other sets of parameters such as properties of the skeletal segments.

## Introduction

A broad goal for research into human biomechanics and motor control is to understand why the musculoskeletal system is built the way it is. One way of trying to understand the functional role of human morphology is to examine large morphological changes present in the fossil record that relate to large changes to human behavior. For example, Roach et al. [1] present interesting evidence that the design of our upper limbs diverged from that of our evolutionary ancestors along with the start of hunting with tools. These design changes resulted in increased power output in our upper limb joints during the throwing of a projectile, which required modification of our shoulder girdle, glenohumeral joint and the supporting ligaments. One reason that over-arm throwing draws the attention of biomechanists is that we are so much better at this movement than our phylogenetic relatives.

A complementary approach to understanding the function of human morphology is to make predictions about the anatomical and physiological characteristics that are optimal for a motor task. The way task performance depends on muscle volume distribution and muscle state parameters —for example muscle length, muscle velocity, and the rate of calcium binding to troponin —is complex and it is hard to predict what are, in fact, the physiological parameters that optimize performance of a task. Using numerical simulation and optimization of a realistic musculoskeletal model, we can address this problem and predict what musculoskeletal design is task optimal. Such an approach yields a human body designed for a single behavior and allows us to test predictions about optimal anatomy and physiology. This approach also allows us to understand how selective pressure on human physiology has resulted in the compromise of singular outstanding performance in one task for successful, albeit not optimal performance across a broad motor repertoire.

In this paper we make a prediction about optimal human morphology for a well-studied and relatively simple human movement, vertical jumping [2, 3]. We investigate the questions: how high could a human jump if muscle distribution was optimized for jumping, and how does the optimized design realize its superior performance? There are many candidate motor behaviors that could be used to study such questions of optimal functional morphology, but vertical jumping is an excellent example task because

- the task goal—jump as high as possible—is unambiguous
- existing models predict performance that well matches empirical data
- muscle models feature realistic muscle parameters about force delivery

While any characteristics of human morphology might be investigated using this approach, we chose here to investigate the redistribution of muscle volume. For the sake of clarity in the analysis we have kept the number of parameters small and considered the effect on vertical jumping of only two parameters per muscle: 1) cross-sectional area and 2) muscle fiber optimum length (note: throughout this text **muscle fiber optimum length** refers to the single value reflecting a given muscle’s optimum length while **muscle length** refers to the time-varying state of a muscle).

## Results and Discussion

**Fig 1:**
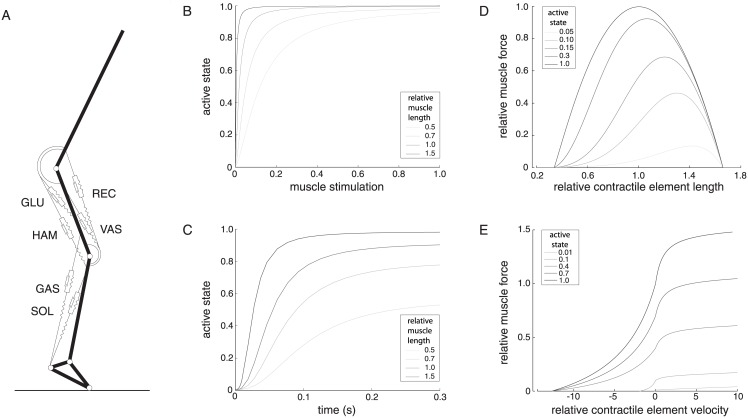
Musculoskeletal model. The muscle model has been used extensively in previous work, and a complete description is documented in [17]. **A**. The body was modeled as a four segment inverted pendulum connected with pin joints. **B-E** Muscle model. **B**. In steady state, the active state (relative amount of bound [Ca 2+] to troponin) of the muscle is a function of muscle stimulation and muscle length. **C**. Active state of the muscle over time, at different isometric muscle lengths. **D**. The force-length relationship is parabolic, the shape and peak of which shifts with the active state of the muscle. **E**. The force-velocity relationship captures the dynamics described in [18] during concentric contraction. The eccentric part of the force-velocity relationship was modeled using a hyperbola and at infinitely high velocity asymptotically approaches 1.5 times isometric force. Muscles are as follows: GLU = gluteus; HAM = hamstrings; VAS = vastus; REC = rectus femoris; SOL = soleus; GAS = gastrocnemius.

We used a four-segment model of human squat jumping. The model includes six lumped leg muscles important for jumping. Since only a single muscle of each was included in the model, we refer to the six muscles as gluteus (glu; all monoarticular gluteals), hamstrings (ham), vastus (vas; all monoarticular vasti), rectus femoris (rec), soleus (sol; soleus and deep plantar flexors), and gastrocnemius (gas). The model has been employed previously in numerous investigations about vertical jumping [3–6], and in particular to model the jumping behavior of skilled male volleyball players, both in terms of performance and in muscle EMG onset times. Hill-type muscles were modeled, the force from which is a function of active state, muscle length, and the muscle fiber velocity (Fig 1). We optimized muscle distribution for maximal human vertical jump height under the constraint that total muscle volume remained constant, since increasing total muscle volume would increase the maximal amount of available actuator work and obviously improve jumping performance. To explore the role of muscle volume in jumping, we investigated the effects of changes to the cross-sectional area and muscle fiber optimum length of each muscle on maximum jump height. Note that if muscle fiber optimum length is changed and tendon slack length is held constant, that muscle’s torque-angle relationship will be altered. Therefore we kept constant, for all models, the joint angle at which isometric muscle force was maximal. This was done by accompanying all changes in muscle fiber optimum length with dependent changes in muscle tendon length. Muscle input was modeled as bang-bang control, so that each muscle’s activity changed from initial to maximal at a single onset time per muscle. Note that this is thus a very simple control scheme but it approximately reflects muscle activation during maximal jumping and has been observed to sufficiently capture jumping behavior from a wide range of starting positions [6]. Note also that in the reference model segment and muscle parameters were based on human anthropometric data [4] and not in any way tuned to maximize vertical jumping. During optimization of muscle volume, the six muscle control signals (onset timings of each muscle) were allowed to vary along with the cross-sectional area and muscle fiber optimum lengths of each muscle.

### How much can jump height be increased by choosing the best muscle fiber optimum lengths and cross-sectionals of leg muscles?

**Fig 2:**
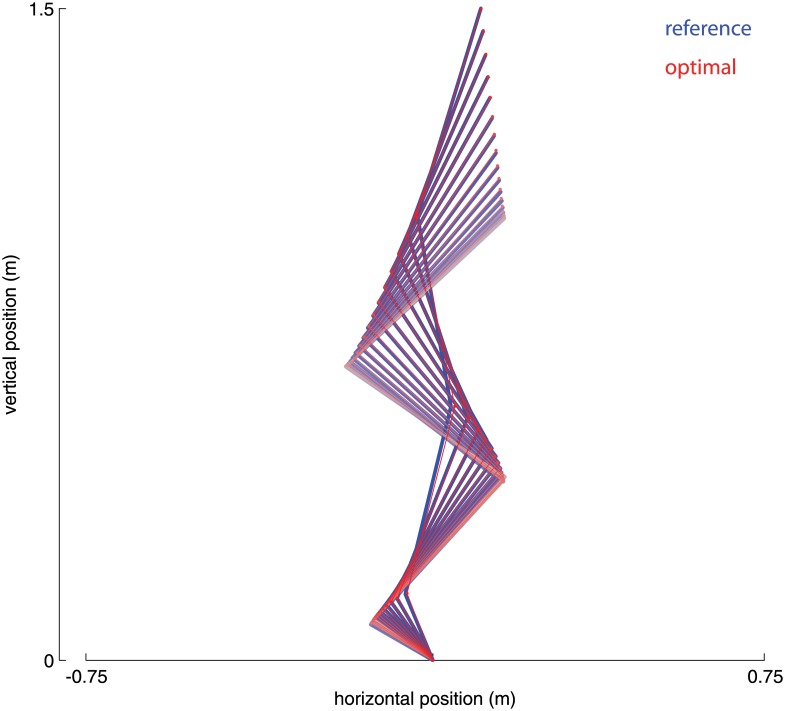
Segment kinematics, sagittal plane view. Plotted are the foot, shank, thigh, and trunk during the push-off for both reference (blue) and optimal (red) models. Segments have been plotted at 5% increments of the push-off phase. Percentages reflect the relative increase in jump height over the reference model. COM = height of center of mass.

The stimulations and muscle properties to maximize vertical jumping were found using a sequential quadratic programming algorithm [7–10] and checked with a custom genetic algorithm [11]. The optimized model produced an increase in jump height of 6.1 cm from 41.2 cm, an improvement of 15%. Fig 2 shows the kinematics of both the reference and optimized models at 5% increments of the push-off phase. When time is normalized to the duration of each push-off, the joint angle trajectories are very similar for optimized and reference models. Thus the optimal solution does not exploit a novel movement pattern unavailable to the reference model, but rather the same movement pattern is performed by the optimal jumper, only faster—in 22 ms (7%) less time.

### Optimal muscle volume distribution: cross-sectional area and muscle fiber optimum length

The simulated optimal jumping models found the optimization of muscle cross-sectional areas to be more efficacious than the optimization of muscle optimum lengths: CSA optimization provided a 4.4 cm (11%) increase in jump height over a baseline of 41.2 cm. Combined CSA and fiber optimum length adjustments allowed for only an additional 1.6 cm increase (4%). When muscle fiber optimum length was altered alone to maximize jump height, a 3.0 cm (7%) improvement was observed. Since the simultaneous optimization of both cross-sectional area and muscle optimum lengths results in nearly the same set of optimal muscles as the optimization for cross-sectional area alone and constitutes the majority of improvement, we focus our remaining analyses on understanding the performance enhancing effects of the changes to muscle cross-sectional area.

### Optimal muscle volume distribution: gastrocnemius, vastus, hamstrings

The optimal distribution of leg muscles (Table 1) shows approximate doublings in the cross-sectional area of gastrocnemius (ankle extensor, knee flexor), vastus (knee extensor) and an approximate 30% increase in hamstrings (knee flexor, hip extensor). These increases occur while reductions in the cross-sectional area are observed in soleus (ankle extensor), rectus femoris (knee extensor, hip flexor) and gluteus (hip extensor).

**Table 1:** Muscle volume and work for both the reference and optimized models.

Muscle	Reference model % total volume	Optimal model % total volume	Reference model muscle work (J)	Optimal model muscle work (J)
Soleus	14	4	77.5	11.4
Gastrocnemius	7	15	39.0	87.3
Vastus	27	52	188.4	353.0
Rectus Femoris	8	0	12.9	0.0
Gluteus	32	12	208.7	75.6
Hamstrings	13	18	109.8	148.4
Total			636.2	675.8

Before addressing why the optimal set of muscles is better for jumping, it is important to understand how much better this set of muscles is compared to other possible distributions. It might be, for example, that the multi-dimensional landscape that relates possible muscle distributions to jump height is flat. If that were true, then many muscle redistributions would result in similar performance.

### Optimal muscle distribution: constrained optimizations to test other potential solutions

To test whether the optimal jumper (Table 1) is only marginally better than many different arrangements all of which achieve similar jump heights, we performed a constrained optimization to find other distinct muscle redistributions, i.e. jumping models using a different set of muscles. The optimal model in Table 1 has large monoarticular knee extensors, and biarticulars that cross the knee and extend the adjacent ankle (gastrocnemius) and hip (hamstrings), and here we explored the performance of distinct distributions. For example, one alternative solution would utilize the analogous muscle redistribution at the hip and involve large monoarticular hip extensors, and biarticulars to extend the knee (rectus femoris) and ankle (gastrocnemius). We therefore performed a constrained optimization to find jumping models with larger gluteus, rectus femoris, and gastrocnemius. Note that it might also be interesting to consider a jumper with a large monoarticular ankle extensor and biarticular muscles extending the knee and hip, but a biarticular ankle flexor/knee extensor does not exist in humans. A second constrained optimization we analyzed was a monoarticular jumper featuring only large soleus, vastus, and gluteus muscles. Taking the results of these two constrained optimizations together provides insight into whether the optimal gastrocnemius-vastus-hamstrings jumper is unique in its ability to improve jump height above the reference model, or merely the best of many similar configurations.

Constrained optimizations revealed that the solution with large gastrocnemius, rectus femoris, gluteus muscles is significantly inferior and achieves only 84% of jump height of the gastrocnemius-vastus-hamstrings model, and happens also to be 8% worse than the reference model. Similarly, the monoarticular jumper is inferior to the optimal model, but in fact performs nearly identically (within 2%) of the reference model. Since both of these constrained optimizations are significantly worse than the optimal gastrocnemius-vastus-hamstrings model, we can be confident that the gastrocnemius-vastus-hamstrings solution is not merely one of several equivalently performing muscle redistributions.

### Optimal muscle volume distribution: why is the gastrocnemius-vastus-hamstrings model optimal?

Below we discuss several possible factors that may contribute to the optimized model’s improved jumping ability to understand 1) how the optimal model acquires its performance improvements and 2) why a given muscle should be included or excluded in the optimal model.

#### Optimal muscle volume distribution: motor efficacy?

At the end of the push-off, jump height is determined by the height of the center of mass and the vertical velocity as the foot leaves the ground. Effective work for vertical jumping is defined as the sum of energies that determine jump height: i.e. the sum of vertical kinetic energy and gravitational potential energy of the center of mass. An example of ineffective work would be velocity of the center of mass in the horizontal direction at the end of push-off. The efficacy ratio is defined as the ratio of effective work to the total work done by the muscles [5]. We can compute total work done on the skeleton for a given set of muscles if we integrate muscle power with respect to time (and account for any energy difference in the elastic tendons) and measure how that amount of muscle work compares to the change in kinetic energy of the center of mass, rotational kinetic energy of limb segments, and gravitational potential energy. It could be that the optimal model achieves its superior performance by increasing this efficacy ratio.

The optimal model does 6% more total work: 636 and 676 J of work are done during push-off for the reference and optimized models respectively. The optimized model jumps 4.4 cm higher, which is equal to an increase of 35.5 J of gravitational potential energy. Since the increase in gravitational potential energy nearly equals the increase in total muscle work, it must be that the optimal model performs the movement with nearly the same motor efficacy ratio. Indeed, it turns out that motor efficacy ratios of the reference and optimal models are nearly identical, and the optimal model only slightly improves motor efficacy ratio over the reference model (88.2% to 87.5% respectively). Given 676 J of mechanical work done by the optimal model, a 0.7% increase in efficacy ratio is equivalent to 4 J more of efficacious work, or 11% of the optimal model’s increase in gravitational potential energy at peak height. Therefore motor efficacy is only a small contributing factor to the increased performance of the optimal muscle distribution.

**Fig 3:**
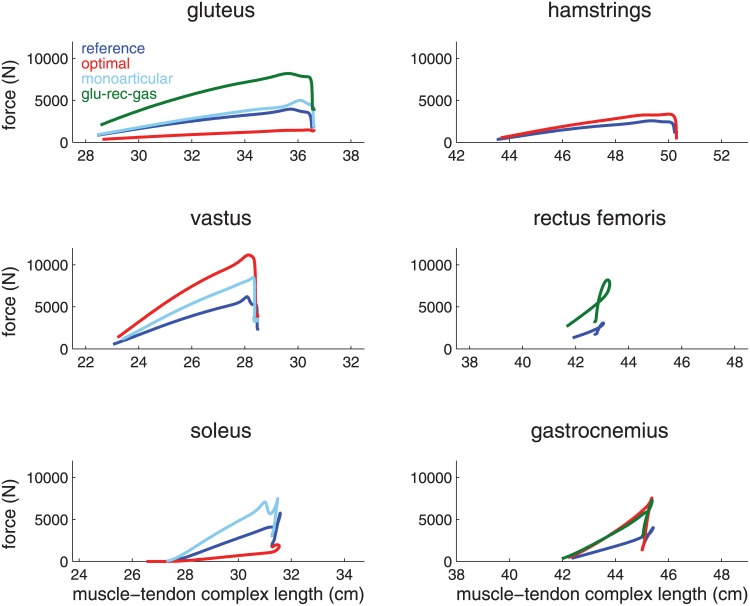
Muscle work for monoarticular model and reference model. The force as a function of muscle-tendon complex length is plotted for each of the muscles in the monoarticular model (dashed lines) and reference model (solid lines). Total muscle volume for both models are held constant. The monoarticular muscles are presented in the left column. glu-rec-gas = gluteus-rectus femoris-gastrocnemius model.

Motor efficacy is also only a small contributing factor to why the monoarticular model is suboptimal. The optimal redistribution of muscle volume into three monoarticular muscles (soleus-vastus-gluteus) jumps within 2% (0.5 cm) of the reference model that has six muscles. The muscles in the monoarticular model deliver slight 1% (7 J) more work but achieve lower motor efficacy (86% versus 87%) than the reference model because the segments have higher rotational kinetic energy (10 J). Fig 3 shows force as a function of muscle-tendon complex length and thus the area under each curve represents the total work done by each muscle.

#### Optimal muscle volume distribution: muscle work per volume

The optimal muscle distribution for jump height by definition has a greater ratio of effective work per total muscle volume (since the numerator, effective work, has increased while the denominator has remained constant). It might be predicted further that the work per volume for each individual muscle also increases. Table 2 lists the work per volume of each muscle for both the reference and optimal models, and normalized by the most efficient muscle (hamstrings).

**Table 2:** Individual muscle work per volume for the reference and optimized models. The first column is the work/volume for each muscle in the reference model (and the second column presents these data normalized by the most efficient muscle, hamstrings). The third column shows the work/volume ratio in the optimal model. The fourth column again presents this volume-specific work of the optimal model normalized by that of the hamstrings. The fifth column shows the percentage by which each muscle is scaled in the optimal model, relative to the reference model (values larger than 1 therefore indicate an increase).

	Reference work per volume (J/L)	Reference normalized	Optimal work per volume (J/L)	Optimal normalized	(Optimal volume) / (reference volume)
Soleus	44.0	0.67	24.9	0.38	0.26
Gastrocnemius	44.3	0.66	46.8	0.71	2.12
Vastus	56.3	0.85	53.9	0.82	1.96
Rectus femoris	13.3	0.2	6.1	0	0
Gluteus	52.2	0.79	50.7	0.77	0.37
Hamstrings	66.0	1	67.1	1.02	1.33

It is evident that optimizing for jump height does not result in increases in the work per volume for individual muscles. There is no clear correlation between work/volume ratio and the scaling parameters. For example, soleus and gastrocnemius have approximately the same volume-specific work, and gluteus and vastus volume-specific work are also comparable. A notable exception is the rectus femoris which has very low work per volume compared to the other muscles.

#### Optimal muscle volume distribution: over-specialization?

**Fig 4:**
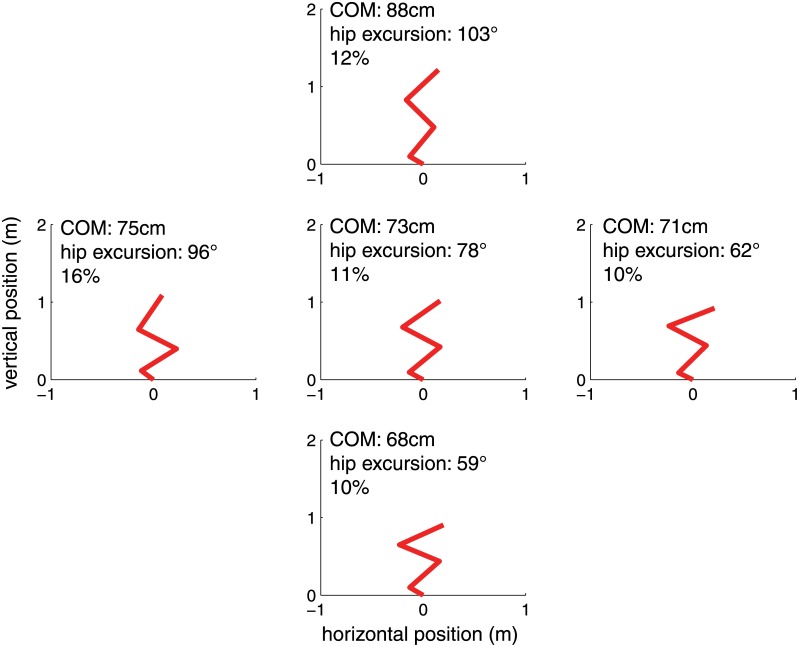
Jump performance from different initial positions. We examined the performance of the model, which was optimized for the center position, at four different initial configurations. The vertical positions vary in the initial center of mass, from 68 cm (bottom) to 88 cm (top). The horizontal positions vary in internal hip angle, from 62 to 96 degrees.

It might be that the optimal model is highly specialized and jumps well at the single position used for optimization (Table 3), but at the cost of general jumping performance, for example when initiated from other initial postures. We examined this possibility by determining the jump heights of the reference and optimal models from four initial positions that differed in 1) the height of the center of mass and 2) the angle between the hip and thigh. We compared the jump heights achieved by both reference and optimal models (Fig 4), and found that in each case the optimal model out-performed the reference model by between 10 and 16%. This finding suggests that the optimal solution is not highly specific and does not achieve a singular performance at the cost of general performance.

**Table 3:** Initial conditions. Initial angles and joint excursion of the optimized jump. Angles noted in degrees.

	Toe	Ankle	Knee	Hip
Initial position	144.8	-97.3	97.9	-102.5
Joint excursion(reference model)	-32.3	61.1	-74.3	75.5

Motor efficacy ratio, individual muscle efficiency, and over-specialization do not clearly explain the source of the optimal model’s performance gain. We next explore other potential factors that define the set of optimal jumping muscles.

### Individual muscles

#### Individual muscles: rectus femoris

The rectus femoris is particularly ineffective in human jumping given its standout low work-volume ratio (Table 2). To understand this result, we consider in Fig 3 the muscle-tendon complex length of the rectus femoris during push-off. Because of approximately equal moment arms at the knee and hip (4.2 vs 3.5 cm, respectively) and very similar joint excursions of the knee and hip during push-off, the rectus stays nearly the same length throughout, changing only 0.8 cm (Fig 3); it therefore can do almost no mechanical work on the skeleton.

#### Individual muscles: gluteus

**Fig 5:**
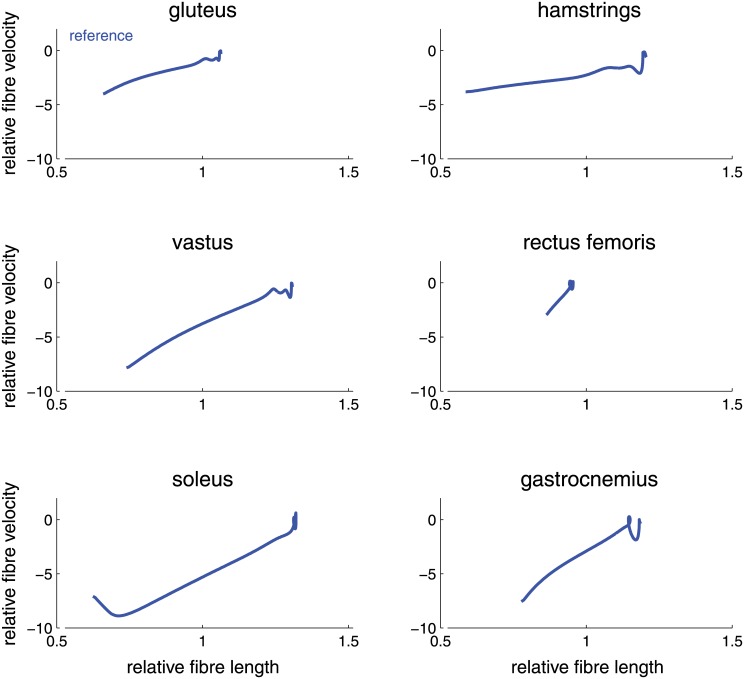
Optimum fiber lengths per second *s*^−1^ and relative muscle length for the reference model muscles. Muscles begin at relatively long length and shorten during push-off. Note that relative muscle length of the gluteus is particularly short at the beginning of push-off.

Above we noted that muscle fiber optimum length affected jump height to a much smaller degree than muscle cross-sectional area. However for the gluteus, fiber optimum length matters significantly. Fig 5 plots the normalized muscle velocity (units s^−1^; negative velocities reflect concentric contraction) as a function of normalized (dimensionless) muscle fiber optimum length in the reference model. A number of muscles begin at similar muscle fiber lengths: gastrocnemius, vastus, and hamstrings begin at long relative lengths, while the gluteus begins at a shorter relative fiber length of approximately 1.1.

The force-length relationship defines relative muscle force to vary approximately quadratically with muscle length (normalized to optimum length, the length at which the muscle delivers maximum force during isometric contraction). Assuming an identical relative velocity profile, the gluteus would deliver more work if the muscle length change was symmetric about the operating range. This would mean that the muscle would need to be at a longer normalized length when push-off begins. There are two ways of increasing the relative length of the gluteus at the start of push-off: 1) move the insertion points of the muscle further away from the joint axis, while keeping muscle fiber length constant or 2) shorten the fiber length of the gluteus such that at the beginning of push-off it is at a longer relative length. This could be done by shortening the muscle fiber length while leaving the tendon slack length unchanged.

Note that the gluteus does not only have a large cross-sectional but also a large length compared to other muscles (Table 4). It is the longest muscle by nearly a factor of 2. Therefore not only does the gluteus operate in a sub-optimal range but it represents a large amount of muscle volume to be potentially reallocated.

**Table 4:** Muscle parameters. Muscle cross-sectional area (CSA), muscle fiber optimum length and tendon element slack length of the reference model.

Muscle	CSA (cm^2^)	Muscle fiber optimum length (cm)	Tendon length (cm)
Soleus	320	5.5	23.6
Gastrocnemius	160	5.5	37.6
Vastus	360	9.3	16.0
Rectus femoris	120	8.1	34.0
Gluteus	200	20.0	15.0
Hamstrings	160	10.4	37.0

#### Individual muscles: alteration of limiting parameters

The optimal distribution of leg muscles for vertical jumping produces a configuration in which muscle volume is gained in the gastrocnemius, vastus, and hamstrings. To test our arguments that explain why other muscle distributions are inferior, one might first perform simulations in which the parameters that make some muscles ineffectual for jumping are added to the list of free parameters, and then determine if these new solutions now admit near-optimal jumping performance. Of course, as more parameters of the musculoskeletal system are freed for optimization, the increased dimensionality will by definition allow for other solutions with similar or superior performance. Instead, to test our predictions we made just two specific alterations to the model:

- The magnitude of the moment arms of the rectus femoris at the knee and hip were changed to match those of the hamstrings at the hip and knee, so that the rectus femoris muscle excursion matches that of the hamstrings.
- The muscle fiber optimum length of the gluteus was shortened to be 80% of its original optimum length while leaving the tendon slack length constant, so that the gluteus begins at a longer relative length.

**Fig 6:**
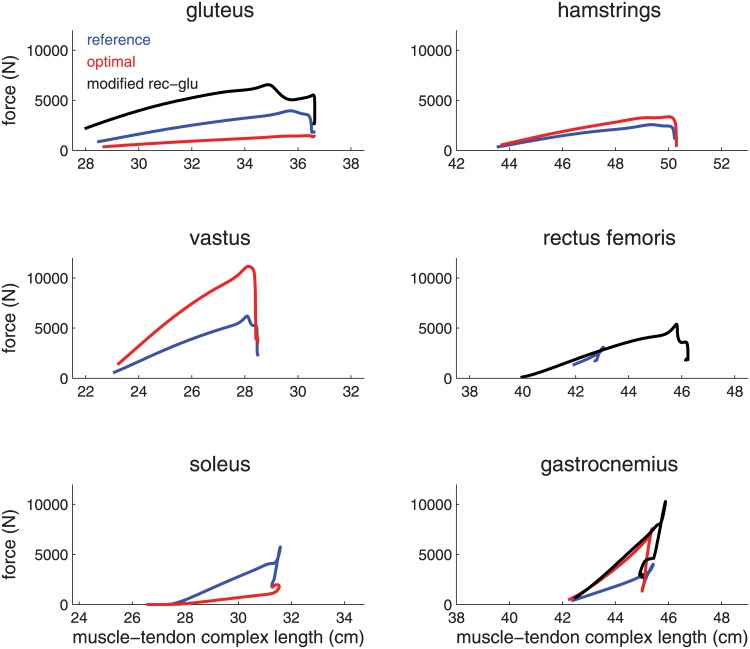
Muscle work for muscles of the reference, optimal, and modified model. The force as a function of muscle-tendon complex length is plotted for each of the six muscles in the reference (solid), optimal (dash-dot), and modified (dotted) model.

The resulting modified model jumps higher than the reference model (44.3 cm vs 41.2 cm, 7%), and when muscle distribution is optimized, is able to jump higher than the optimal redistribution listed above (48.4 cm vs 45.6 cm, 6%). The best redistribution of muscle volume is still a gastrocnemius-vastus-hamstrings muscle redistribution but its superiority over a gastrocnemius-rectus femoris-gluteus model is only 1.5 cm (compared to a 6.9 cm difference in the unmodified model optimizations above). Fig 6 depicts muscle force as a function of muscle-tendon complex length for the reference model and modified model which demonstrates the overall solution. The differences in rectus femoris and gluteus are very evident, and provide support for our arguments about muscle optimality.

### General discussion

We used a model-based approach to understand how muscle morphology contributes to motor performance. We investigated the optimal muscle distribution for vertical jumping in humans. We found that the optimal configuration of gastrocnemius-vastus-hamstrings jumps 15% higher, and does so while the joint angles over time (where time is normalized to the duration of push-off) during the push-off were very similar to the reference model. Thus, the optimal model does not exploit novel movement patterns to gain performance. The optimal model has higher power output and delivers more work to the skeleton in a shorter push-off phase. We considered and ultimately rejected a number of possible explanations for the optimal model’s performance —motor efficacy ratio, individual muscle work-volume, and over-specialization. On the basis of further energetics analyses we argued that the optimal jumping model features a muscle volume distribution best equipped to deliver work during the push-off while removing muscles that have less capacity to deliver work. We tested our energetics analysis by running specific optimizations on a modified model that produced a near optimal model with distinct muscles, thereby providing support for our analysis. This approach outlines a novel and interesting approach to understanding the role of functional morphology in motor performance.

In this study we chose to focus our analysis on the relation between muscle volume and task performance. By focusing on this small parameter set we were able to understand how those cross-sectional area and length contribute to task performance. Importantly, insight was not limited to the parameters that were freed to be optimized: we also demonstrated that changes to other variables, such as the moment arms of the rectus femoris and the gluteus force-joint angle relationship, were able to convert poor muscle redistributions for jumping (those including large rectus femoris and gluteus muscles) into excellent ones. It might follow that one extension of this approach would continue to explore increasing numbers of free musculoskeletal parameters for jumping. Of course, by increasing the dimensionality of an optimization problem we will by definition produce many other solutions with similar or superior performance. Furthermore, our ability to understand the optimal solution greatly decreases as the number of free parameters increases. This might be acceptable depending on the research question. For example, it might be interesting to explore how natural selection produces many distinct solutions to the same biomechanical problem when many morphological parameters are available. The goal of this paper was rather to first determine how relatively good normal human musculature is to an optimized design, and then understand the optimal biomechanical properties for a motor task by studying the advantages in terms of mechanics and energetics. This approach could in principle be used to explore the basis behind biomechanical features for any given motor task, and might contribute to investigations into how variation in human task performance can be explained by the combination of optimal design and optimal control.

The reference jumping model used in this study has been previously employed to model the average jumping performance of an athletic subpopulation of human males. Of course the degree of improvement that optimization can yield is in part determined by the population used as a reference model. It might therefore be interesting to investigate the relative gains made by optimizing physiological parameters based on populations of varying skill and physique. Finally, while it is conceivable that physiological parameters of elite athletes could be predicted by constrained optimization and in turn help to validate a forward simulation model, such explorations are beyond the scope of the current study.

This study suggests that the functional morphology of human musculature is reasonably close to the optimal distribution for maximum vertical jumping. This result might be surprising, given that we do not appear to require jumping to evade predators, acquire food, or attract mates. While near-optimality might suggest that the human system is in fact optimized to jump, perhaps more plausibly other motor tasks such as locomotion simply require musculature for leg extension movements that are similar to vertical jumping. Nevertheless, some muscles in the existing model do not seem essential for jumping behavior, such as the muscles rectus femoris and gluteus. While EMG studies suggest they both are used by the nervous system during jumping [12] and stimulated near-optimally for different jumping start positions [3], their existence and characteristics may reflect a prominent functional role in other human movements. This paper demonstrates a model-based approach that uses predicted optimal physiology to address questions about functional morphology.

## Materials and Methods

Vertical squat jumps were simulated using a musculoskeletal model capable of successfully reproducing human vertical jumps [6, 13]. It was comprised of four body segments, actuated by six major muscle-tendon complexes (MTCs) of the human lower limbs. The musculoskeletal model’s lengths and inertias are based on anthropometrics of skilled male volleyball players. Optimal fiber lengths are based on sarcomere numbers of human cadaver material. Each MTC was represented by a Hill-type muscle model, comprised of a contractile element (CE), series elastic element (SE) and parallel elastic element (PE). Forces of SE and PE scaled quadratically with elongation, while CE force depended on fiber length, the time-derivative of fiber length, and active state (Fig 1). Maximum muscle fiber contraction velocity was proportional to muscle fiber optimum length. Active state had first order dynamics defined by the neural stimulation to the muscle, which during jumping varied according to a single onset time per muscle.

Initial stimulation levels were set such that the model was in equilibrium. During the jump, stimulation of each muscle rose directly to maximal at a single stimulation onset time. The combination of stimulation onset times that maximized the height achieved by the center of mass was found using sequential quadratic programming [7–10] and checked using a genetic algorithm [11]. The total muscle volume—muscle fiber optimum length times cross-sectional area—was kept constant.

By redistributing muscle volume, we also move muscle mass. In models using segment lumping of the skeleton and muscle mass, there are limitations to the degree to which the lumped mass accurately reflects true dynamics [14]. Nonetheless, to account for variations in muscle mass we performed the following segment mass and moment of inertia calculation during optimization. We recomputed the mass and moments of inertia assuming a specific tension of 250000 N m^−2^[15] and density of 1059 kg m^−3^ [16]. This results in a total muscle mass of 13.4 kg. This muscle mass was distributed over the segments in proportion to the total muscle volume assigned to the muscles located in each segment. In addition, the moment of inertia of each segment was adapted in proportion to the change in segment mass. However, it turns out that optimizations with and without the adjusted segment masses and inertias result in very similar solutions. Because solutions did not depend on mass redistribution, it is not the case that optimal solutions significantly benefit from mass being positioned advantageously. This is likely because the muscles in the lower extremities make up only about 15% of the total mass of the human body.
